# Heterogeneity in glycan composition on the surface of HIV-1 envelope determines virus sensitivity to lectins

**DOI:** 10.1371/journal.pone.0194498

**Published:** 2018-03-26

**Authors:** Muzafar Jan, Chitra Upadhyay, José Alcami Pertejo, Catarina E. Hioe, Sunil K. Arora

**Affiliations:** 1 Department of Immunopathology, Post Graduate Institute of Medical Education & Research, Chandigarh, India; 2 Department of Medicine, Division of Infectious Diseases, Icahn School of Medicine at Mount Sinai, New York, New York, United States of America; 3 James J. Peters VA Medical Center, Bronx, New York, United States of America; 4 Imunopatologia Del SIDA, Centro Nacional De Microbiologia, Instituo De Salud Carlos III, Madrid, Spain; Deutsches Primatenzentrum GmbH - Leibniz-Institut fur Primatenforschung, GERMANY

## Abstract

Lectins that target *N*-glycans on the surface of HIV-1 envelope (Env) glycoprotein have the potential for use as antiviral agents. Although progress has been made in deciphering the molecular details of lectin and Env glycan interaction, further studies are needed to better understand Env glycan heterogeneity among HIV-1 isolates and its influence on virus-neutralization sensitivity to lectins. This study evaluated a panel of lectins with fine specificity for distinct oligosaccharides and assessed their ability to inhibit infection of HIV-1 viruses known to have differing sensitivity to anti-HIV Env antibodies. The results showed that HIV-1 isolates have different sensitivity to lectins specific for α1-3Man, α1-6Man, and α1-2Man binding lectins. Considering that lectins exclusively recognize the oligosaccharide components of virus Env, these data suggest that glycan heterogeneity among HIV-1 isolates may explain this differential sensitivity. To evaluate this further, chronic and acute viruses were produced in the presence of different glycosidase inhibitors to express more homogenous glycans. Viruses enriched for α1-2Man terminating Man_5-9_GlcNAc_2_ glycans became similarly sensitive to α1-2Man-binding lectins. The α1-3Man- and α1-6Man-binding lectins also were more potent against viruses expressing predominantly Man_5_GlcNAc_2_ and hybrid type glycans with terminal α1-3Man and α1-6Man. Furthermore, lectin-mediated inhibition was competitively alleviated by mannan and this effect was augmented by enrichment of mannose-type glycans on the virus. In addition, while Env of viruses enriched with mannose-type glycans were sensitive to Endo-H deglycosylation, Env of untreated viruses were partially resistant, indicating that HIV-1 Env glycans are heterogeneously comprised of complex, hybrid, and mannose types. Overall, our data demonstrate that HIV-1 isolates display differential sensitivity to lectins, in part due to the microheterogeneity of *N*-linked glycans expressed on the surface of the virus Env glycoprotein.

## 1. Introduction

With nearly 2 million new HIV-1 infections each year, the development of an HIV-1 vaccine remains a top public health priority. The envelope glycoprotein (Env) is the only viral antigen present on the surface of HIV-1 virions. HIV-1 Env mediates the initial steps of viral attachment and entry into host CD4 T cells using the receptor CD4 and the co-receptors CCR5/or CXCR4.

Env is decorated with a dense covering of *N*-glycans, often called the “glycan shield,”; this shield affects the structural, biological, and immunological properties of Env. Consequently, the *N*-glycans influence virus-cell interactions and Env recognition by the immune response [1–6]. The glycan shield contributes approximately 50% of Env molecular mass, covering most of the Env surface. While these glycans mask the conserved neutralizing antibody epitopes on HIV-1 Env [1, 7, 8], they can also be targeted by broadly neutralizing antibodies (bnAbs) that are generated in a fraction of HIV-1-infected individuals after many years of infection [9, 10]. Indeed, the majority of recently isolated bnAbs have been shown to recognize glycan-bearing epitopes [8, 11–13]. These bnAbs are attractive anti-HIV-1 agents for passive transfer therapy, but their activity is influenced by the types of glycans expressed by the virus Env [13–15]. Researchers have been working to identify the glycosylation profile and determine the extent of oligosaccharide microheterogeneity at each potential glycosylation site on the most native forms of Env [16–18]. However, the precise characterization of HIV-1 Env glycosylation is difficult, because of the extremely heterogeneous nature of glycoforms on *N*-glycan in general [19–21] and the numerous potential glycosylation sites on HIV-1 Env. Each Env protomer contains 25 to 30 potential *N*-linked glycosylation sites (PNGS), which add up to ~90 PNGS on a single trimeric Env spike. This diversity is further increased by variable occupancy and microheterogeneity of glycoforms at each PNGS for an individual Env molecule [22]. Glycan composition is further complicated by structural constraints of Env trimerization, virus diversity, and quasi-species at different stages of infection, as well as by the types of host cells producing the virus [23–26]. Nonetheless, Env glycan heterogeneity plays a significant role in determining Env biological and immunological properties [27, 28]. Evidence is also accumulating that Env *N*-glycan composition controls virus capture by dendritic cells via C-type lectin receptors, which affects whether virus is degraded for antigen presentation or transmitted to CD4 T cells [29–31].

To further examine *N*-glycan heterogeneity on Env of different HIV-1 isolates, we utilized lectins that display high specificity for particular oligosaccharide structures [32–35]. Lectins have been shown to inhibit HIV-1 infection by binding directly to virus Env glycans, thereby causing disruption of receptor-induced conformational changes, inhibition of membrane fusion, and blocking of DC-SIGN binding [31, 36–39]. The presence of lectins drives virus escape associated with loss of multiple glycosylation sites, unmasking potential immunogenic epitopes and increased virus susceptibility to the immune response [40–43]. Of note, the predominant *N*-glycans on HIV-1 Env are oligomannose-type; only a small fraction is composed of complex-type glycans [16, 26]. Accordingly, the majority of lectins with potent anti-HIV activity are specific for mannose residues, especially terminal mannoses on the D1, D2, and D3 arms of the core glycan site; this specificity is similar to that of broadly neutralizing monoclonal antibody 2G12 [34, 44–47]. Anti-HIV lectins typically have IC_50_ values in the nanomolar to picomolar range, and different strategies like oligomerization and domain swapping have been employed to increase the number of glycan-binding sites and further optimize their avidity [34, 47–49]. Nonetheless, antiviral activity of lectins in the context of diverse glycosylation profiles found on different HIV-1 isolates has not been fully explored.

In the current study, we sought to evaluate the sensitivity of different HIV-1 viruses to lectins with fine carbohydrate specificity and to examine the extent to which the glycan heterogeneity of virus Env modulates virus sensitivity to inhibition by lectins. To this end, we selected a panel of lectins with diverse sugar specificities, ranging from purely oligomannose-specific to complex-type glycan-specific. We tested these lectins against viruses with reduced or no *N*-glycan heterogeneity by producing viruses in the presence of glycosidase inhibitors. The data showed differential sensitivity of viruses to mannose-binding lectins, and reducing glycan heterogeneity rendered the viruses more similarly sensitive to these lectins. The data provide evidence for Env glycan heterogeneity on HIV-1 viruses that affects their sensitivity to lectin-mediated inhibition. Data from this study offer valuable information about potential application and limitation of lectins as antiviral agents against HIV-1.

## 2. Materials and methods

### 2.1 Cell lines, lectins, and plasmids

TZM-bl cell line was obtained through the NIH AIDS Research and Reference Reagent Program (ARRRP), contributed by J. Kappes and X. Wu [50]. Cell lines 293T/17 and 293S GnTi^-/-^ were obtained from the American Type Culture Collection (ATCC). All cell lines were maintained in Dulbecco’s modified eagle medium (DMEM; Lonza) containing 10% heat inactivated fetal bovine serum, penicillin/streptomycin (100U/mL), 1M HEPES, and L-glutamine. Cell monolayers were disrupted at confluence by treatment with 0.25% trypsin in 1 mM EDTA. All lectins were purchased from Vector Labs, Sigma, and Cayman Chemicals, and were checked for purity using SDS-PAGE and cell toxicity based on luminescent cell viability assay (CellTiter Glo- Promega). All plasmids were obtained from NIH ARRRP [51–53].

### 2.2 Pseudovirus preparation

Plasmids expressing Env of Bal.01, Bal.ec1, JRFL.JB, REJO4541.67, and SF162.LS for generating pseudoviruses were obtained from NIH ARRRP. Pseudoviruses were produced by co-transfection of HEK 293T cells or HEK 293S GnTI^-/-^ cells with HIV-1 *rev*- and *Env*-expressing plasmids, and pNL4-3ΔEnv or pSG3ΔEnv plasmids, using jetPEI transfection reagent (Polypus-transfect SA). Glycan-modified pseudoviruses were generated as described above in the presence of 25μM kifunensine or 20μM swainsonine. Supernatants were harvested after 48 to 72 h and clarified by centrifugation and 0.45 μm filtration. Single-use aliquots were stored at -80°C. Virus titration was performed on TZM-bl cells, as described previously [52].

### 2.3 HIV inhibition assay

HIV inhibition was measured using a luminescence-based β-galactosidase assay (Beta Glo, Promega) with TZM-bl target cells [54]. Serial dilutions of lectins were added to the virus at 200 TCID_50_ in half-area 96-well plates (Costar) and incubated for designated time periods at 37°C. TZM-bl cells were then added along with DEAE-Dextran (10 μg/ml; Sigma) and incubated for 48 h. Each condition was tested in duplicate or triplicate. Assay controls included replicate wells of TZM.bl cells alone (cell control) and TZM-bl cells with virus alone (virus control). Percentage of inhibition was determined on the basis of virus control under the specific assay conditions. Virus input corresponded to titrated stocks yielding 200 TCID_50_ in RLUs.

### 2.4 Mannan competition assay

Mannan competition assay was performed with serially 2-fold-diluted lectins GRFT and GNA. The lectins were used at a concentration 10 times the IC_50_ of the lectins GRFT (10 μg/mL) and GNA (250 μg/mL) to attain approximately 100% inhibition of glycan-modified viruses. Mannan from *Saccharomyces cerevisiae* (Sigma-Aldrich) was added at 10 mg/mL incubated for 1 h at 37°C. Untreated and glycan-modified viruses (JRFL.JB) were added at TCID_50_ before the addition of TZM-bl cells and incubated for 48 h. The cells were harvested, and virus inhibition was calculated and compared with virus inhibition with no mannan.

### 2.5 Measurement of infectivity of glycan-modified virus

Virus stocks were titrated in TZM-bl cells, and the TCID_50_ was calculated as discussed above. The p24 of the virus stock was determined by enzyme linked immunosorbent assay using HIV-1 p24 ELISA kit (PerkinElmer, USA). Equal amounts of CA-p24 (5 ng/mL) from untreated and glycosylation-modified viruses were used to infect TZM-bl cells for 48 h in the presence of 10 μg/mL DEAE-Dextran and RLUs calculated per nanogram of p24. All experiments were performed in triplicate.

### 2.6 Measurement of Env incorporation into virus particles

Viruses were concentrated from culture supernatants using Lenti-X Concentrator (Clontech-CA), subjected to SDS-PAGE under a reduced condition, blotted onto polyvinylidene difluoride membrane (PVDF), and detected using anti-gp120 mAb cocktail or anti-p24 mAb in a Western blot assay. Equal amounts of viruses (based on p24 contents) were analyzed. In parallel, a known amount of recombinant gp120 JRFL protein (Immunotech) was used as control. The relative amount of Env content was calculated in comparison with standard gp120 by analysing Env bands with ImageLab software (BioRad). The relative amount of Env was quantitated to yield nanograms of Env per nanograms of p24 and expressed relative to untreated virus (set to 100%).

### 2.7 Enzymatic deglycosylation of HIV-1 Env

This assay was performed as described by Raska, et al. [45]. Briefly, viruses were concentrated using 100-kDa Amicon filter (Millipore) or Lenti-X Concentrator (Clontech-CA), and the amounts of Env and p24 in the virus stocks were measured. Virus samples with an equal amount of Env were treated with endo-*N*-glycosidase H (Endo H) from *Streptomyces plicate* that removes selectively mannose- and hybrid-type glycans or with peptide-*N*-glycosidase F (PNGase F) from *Flavobacterium meningosepticum* to remove all glycans. Digestion products were then subjected to SDS-PAGE, blotted onto polyvinylidene difluoride membrane, and detected using an anti-gp120 mAb cocktail. ImageLab software was used for the analysis and quantitation of the blots.

### 2.8 Statistical analysis

All data analysis was performed using S-Plus 6.1 (Insightful Corp.) or GraphPad Prism 6. Unpaired t-tests were performed to compare viral infectivity and Env incorporation between glycan-modified and untreated viruses.

## 3. Results

### 3.1 Differential sensitivity of HIV-1 strains to lectins

To study the glycosylation profile of Env of different HIV-1 strains, we utilized lectins that bind to highly specific oligosaccharide moieties present on particular types of *N*-glycans (Table 1) and compared their ability to inhibit virus infection. We tested 5 HIV-1 pseudoviruses expressing clade B Env with different sensitivity to neutralizing antibodies: SF162.LS (tier 1a), BaL.01 and BaL.ec1 (tier 1b), REJO4541.67 (tier 2, acute), and JRFL.JB (tier 2, chronic) produced in HEK 293 T cells. The Envs of these viruses contained 25, 28, 28, 29, and 28 PNGS, respectively.

**Table 1:** Lectins and their oligosaccharide and N-glycan type specificity.

Lectin	Oligosaccharide Specificity	Glycan types
*Galanthus nivalis* agglutinin (GNA)	-Manα(1–3)Man	Man5/6
*Hippeastrum hybrid* agglutinin (HHA)	-Manα(1–6)Man	Man5/6
*Griffithsia* sp. (GRFT)	-Manα(1–2)Man	D1, D2 or D3 arm of Man8/9
Cyanovirin-N (CV-N)	-Manα(1–2)Man-α(1–2)Man -Manα(1–2)Man	D1 or D3 arm of Man8/9
*Scytonema varium* (SV-N)	-Manα(1–2)Man-α(1–6)Man-α(1–6)	D3 arm of Man9
*Canavalia ensiformis* (Con A)	Man > Glc > GlcNAc Highest binding affinity to Manα(1–3)-[Manα-(1–6)]Man trisaccharide core of N-Glycans	High mannose glycans Hybrid and mannose glycans Complex glycans
*Phaseolus vulgaris* agglutinin (PHA-E)	Galβ1-4GlcNAcβ1-2Man	Complex Glycans
*Lens culinaris* agglutinin (LCA)	α(1–6) linked fucosylated N-linked glycans	Complex glycans

We found that HIV-1 strains displayed differences in sensitivity to lectins (Table 2), similar to that seen with antibodies, with tier 1 viruses more sensitive to lectins than were tier 2 viruses. Hence, the tier 1a virus SF162 was the most sensitive to all lectins as a whole, whereas the tier 2 acute virus REJO was the most resistant. Tier 1b BaL and tier 2 chronic JRFL viruses were intermediate, although BaL was more sensitive than JRFL. This differential sensitivity was observed even though the lectins targeted surface-accessible N-glycans present on Env of the different viruses. All lectins showed no cytotoxicity at the concentrations used.

**Table 2:** Differential sensitivity of HIV-1 viruses to lectins.

		Man_7-9_ GlcNAc_2_	Man_7-9_ GlcNAc_2_	Man_7-9_ GlcNAc_2_	Man_5-6_ GlcNAc_2_	Man_5-6_ GlcNAc_2_	Man+Com	Com	Com	Range		
Ab sensitivity	Viruses	GRFT	CV-N	SV-N	GNA	HHA	Con A	PHA-E	LCA	Mean	Min	Max
Tier 1a	SF162	0.001	0.125	3.023	0.501	1.574	0.861	>50	>50	**0.97**	0.001	3.02
Tier 1b	Bal.01	0.001	0.050	2.988	1.514	9.081	0.349	>50	>50	**2.40**	0.001	9.41
Tier 1b	Bal.ec1	0.008	0.200	9.609	2.786	4.022	1.357	>50	>50	**3.00**	0.008	9.61
Tier 2 (chronic)	JRFL	0.002	0.076	17.600	10.550	26.960	0.121	>50	>50	**9.25**	0.002	26.96
Tier 2 (T/F)	REJO	0.032	0.202	23.900	11.710	48.370	3.080	>50	>50	**14.55**	0.032	48.37
	**Mean**	**0.01**	**0.13**	**11.42**	**5.44**	**18.07**	**1.12**	**>50**	**>50**			
			**<0.1**		**0.1–1**		**1–10**		**>10**			

IC_50_ values (μg/mL) of lectins are shown for tier 1a, tier 1b, and tier 2 viruses against lectins differing in their oligosaccharide recognition specificity. Virus inhibition assay was performed in duplicate and repeated 4 times. Averages of all experiments are shown. Man = high-mannose glycan. Com = complex glycans.

**Fig 1:**
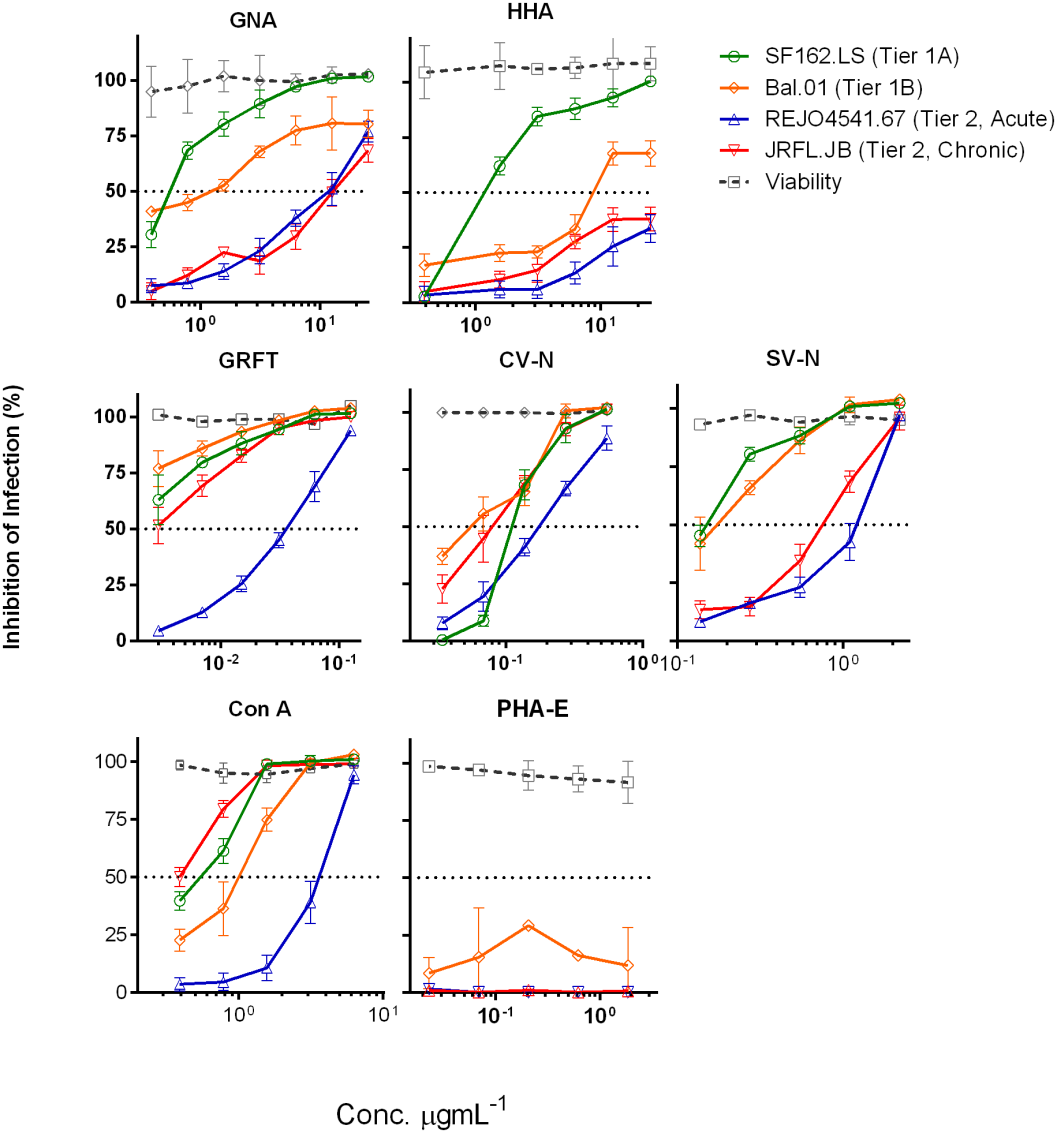
Virus inhibition by lectins that differ in oligosaccharide specificity. Lectins were tested to block infection of HIV-1 pseudoviruses SF162.LS (tier 1A), Bal.01 (tier 1B), JRFL.JB (tier 2, chronic), and REJO4541.67 (tier 2, acute) in TZM-bl cells. Lectins were serially diluted, added to viruses (200 TCID_50_), and incubated for 1 h at 37°C before addition of TZM-bl cells. After 48 h, virus infection was measured by β-galactosidase activity. Cell viability was measured in parallel using CellTiter Glow kit (Promega). Experiments were performed four times; averages and standard deviations from all experiments are shown.

Interestingly, most lectins in this panel were able to attain 100% virus inhibition in a dose-dependent manner, although there were considerable differences in their antiviral potency (Fig 1, Table 2). High-mannose-binding lectins GRFT and CV-N, which recognize terminal α1-2Man residues similar to 2G12 antibody on Man_9_GlcNAc_2_ structures, were the most potent with mean IC_50_ of 0.01 and 0.13 μg/mL against the 5 tested viruses. CV-N was less potent overall, but this lectin inhibited all 5 viruses, including the most resistant REJO virus, with more similar IC_50_ values than GRFT and other lectins. The α1–3 and α1–6 mannose-binding lectins such as GNA and HHA were less effective with mean IC_50_ of 5.44 and 18.07 μg/mL. These results implicate a relative abundance of Man_9_GlcNAc_2_ over Man_5-6_GlcNAc_2_ glycans on HIV Env. Nonetheless, SV-N was much less potent than GRFT and CV-N; this lectin recognizes Man_9_GlcNAc_2_ but displays the narrowest specificity, targeting only the oligosaccharide structure at the D3 arm of Man_9_ GlcNAc_2_. Complex glycan-binding lectins LCA and PHA-E did not inhibit the viruses at the concentration range tested. On the other hand, Con A, a lectin with broader specificity capable of binding of all 3 glycan types, had an intermediate potency relative to lectins highly specific for high mannose or complex glycans.

### 3.2 Effect of *N*-glycan modification on Env molecular mass and virus production

**Fig 2:**
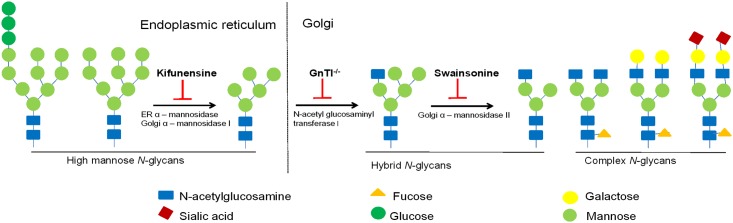
Glycan biosynthetic pathway and its inhibitors. N-linked glycans are transferred *en bloc* onto the nascent peptide after the peptide emerges from the ribosome in the endoplasmic reticulum (ER). The immature high-mannose structure is trimmed by glycosidases and subsequently processed to form hybrid- and complex-type glycans. Kifunensine is a drug inhibitor of the ER and Golgi mannosidase I, thus arresting glycosylation at Man_9_GlcNAc_2_. Production of glycoproteins in GnTI-deficient cells, on the other hand, resulted in accumulation of the Man_5_GlcNAc_2_ structure. Swainsonine inhibits mannosidase II in the Golgi that is required for the maturation of high mannose and hybrid glycans into complex glycans.

To demonstrate whether glycan heterogeneity contributes to differences in virus sensitivity to lectins, viruses were produced to express Env enriched with more homogenous glycans by using drugs that inhibit various stages of the glycosylation biosynthetic pathway or in mutant cell lines lacking GlcNAc transferase I, thus preventing maturation of Man_5_GlcNAc_2_ precursor into hybrid and complex glycans (Fig 2). The impact of Env glycan modification on the biological properties of virus has been examined previously, with inconsistent results [4, 6, 23]. Hence, we first verified alterations in *N*-glycan composition on virus Env prior to testing the viruses in lectin inhibition experiments. We then evaluated the relative production of viruses with vs without glycan alterations.

**Fig 3:**
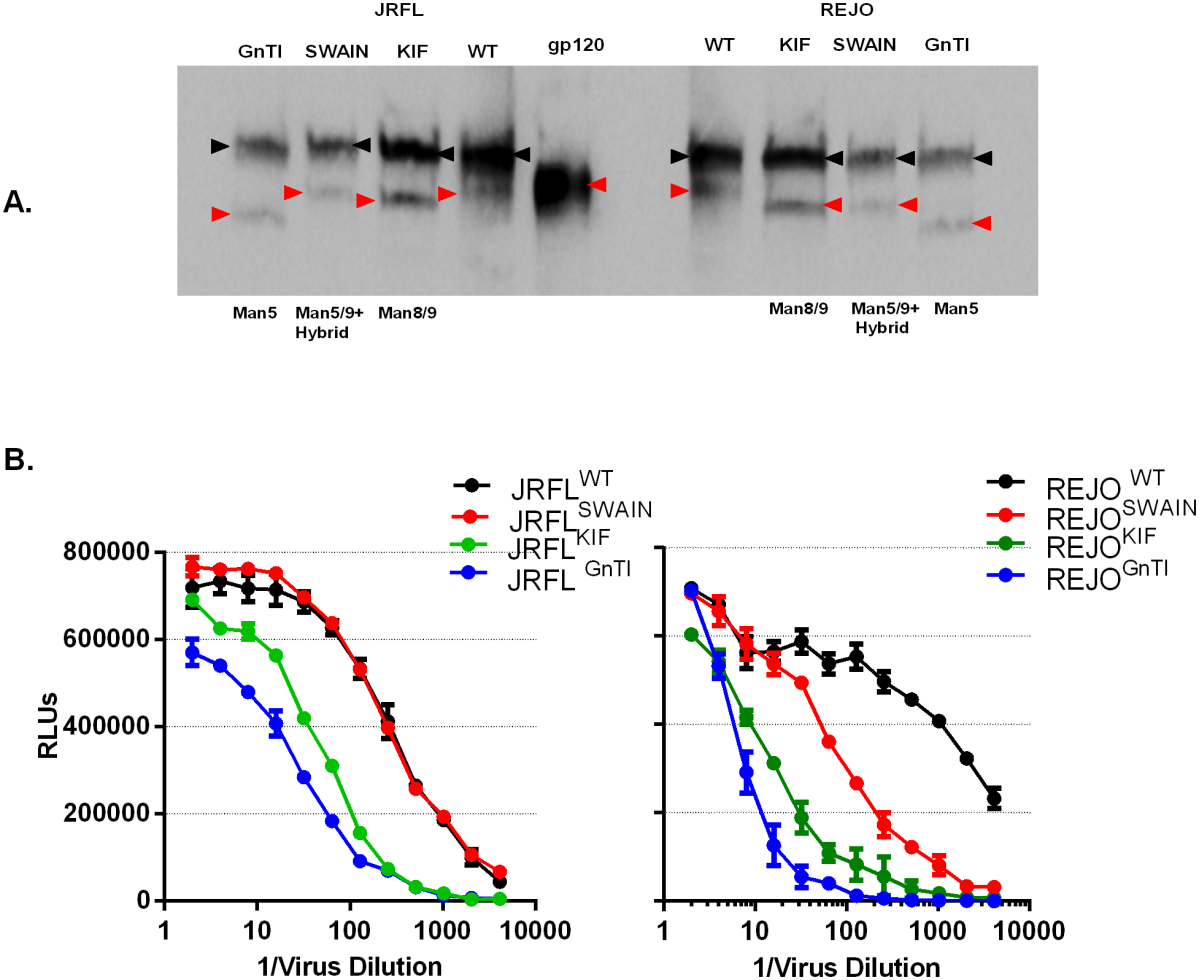
Effects of *N*-glycan modification on Env molecular mass and virus infectivity. Viruses were produced by transient transfection of 293S GnTI^-/-^ cells or 293T cells in the presence or absence of kifunensine or swainsonine. A) Relative molecular weight of Env as measured by SDS-PAGE (reduced condition) and Western blot. Oligosaccharide compositions predominant in the different glycan-modified virus preparations are indicated below the blots. Cleaved Env gp120 and uncleaved Env gp160 are indicated by red and black arrows, respectively. B) Production of REJO4541.67 and JRFL.JB viruses under the different conditions. Culture supernatants containing viruses were serially diluted 2-fold and added to TZM-bl cells. After 48 h, cells were lysed, and β-galactosidase activity was measured with a luminescence substrate. Data represent averages and standard deviations of 2 independent experiments.

Virus production in the presence of kifunensine or swainsonine or in the GnTI-deficient cell line resulted in Env enrichment of Man_5-9_GlcNAc_2_-containing glycans, with an absence of complex glycans. Indeed, when we compared Env from JRFL and REJO viruses produced with glycosidase inhibitors and in GnTI-deficient cells, we found their migration on SDS-PAGE to differ from that of Env of untreated viruses (wild type, WT), indicating molecular weight changes (Fig 3A). Envs of JRFL^WT^ and REJO^WT^ had the highest molecular mass. JRFL^KIF^ and JRFL^SWAIN^ Envs produced in the presence of kifunensine or swainsonine had slightly lower molecular mass than JRFL^WT^. REJO^SWAIN^ and REJO^KIF^ Envs also displayed similar alterations. Env from viruses produced in GnTI^-/-^ cells (JRFL^GnTI-/-^ or REJO^GnTI-/-^) showed the lowest molecular mass. These molecular size changes were consistent with enrichment of Man_5-9_GlcNAc_2_- and hybrid-type glycans on viruses produced in the presence of kifunensine and swainsonine respectively, and with accumulation of mainly Man_5_GlcNAc_2_ glycans in viruses produced in GnTI^-/-^ cells. In addition to greater glycan heterogeneity, the presence of complex glycans could further retard migration of wild type Env in SDS-PAGE, due to negatively charged sialic acid residues. In these Western blots, we also detected prominent uncleaved Env gp160 bands in all virus samples; this might be due to reduced cleavage efficiency when large amounts of Env were produced under a strong artificial promoter as used in our pseudovirus expression system [55].

We subsequently determined whether viruses were produced under different conditions to similar levels. Virus-containing culture supernatants were harvested at 48h after transfection, diluted serially, and added to TZM-bl cells. Glycan-modified viruses had reduced TCID_50_ as compared with their wild-type untreated counterparts, but overall virus production was not drastically altered. Virus production in GnTI^-/-^ cells yielded the lowest TCID_50_, followed by viruses produced in the presence of kifunensine (Fig 3B). REJO^SWAIN^ production also was lower than that of REJO^WT^, whereas JRFL^SWAIN^ was comparable to its wild type.

### 3.3 Effect of N-glycan modification on viral infectivity

**Fig 4:**
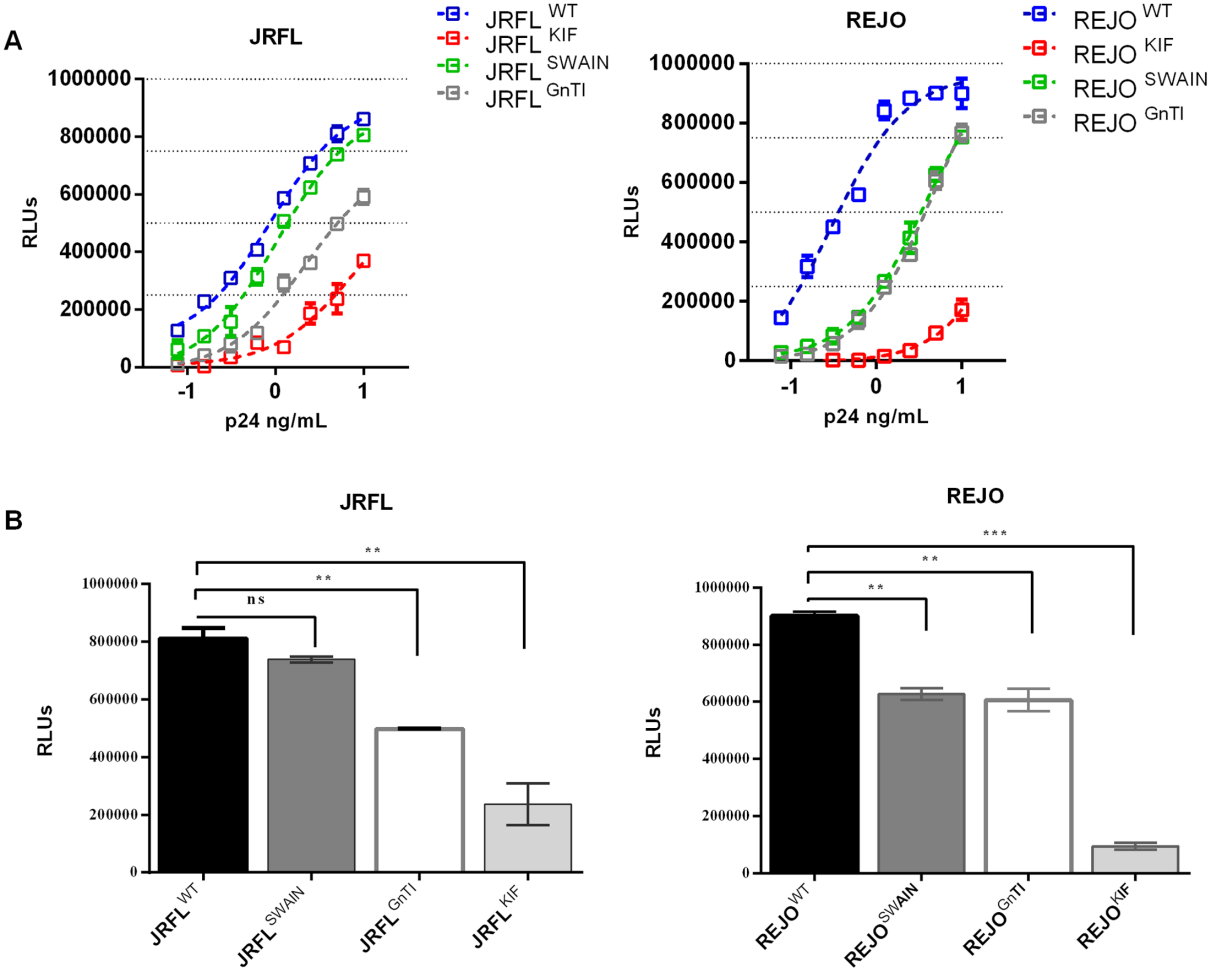
Effect of *N*-glycan modification on HIV-1 infectivity. A) Viruses produced in 293T cells with different glycosidase inhibitors (kifunensine or swansonine) or in 293S GnTI^-/-^ cells were titrated as a function of CA-p24 content and their infectivity in TZM-bl reporter cells was measured after 48 h by β-galactosidase activity. B) Statistical analyses were performed to compare infectivity of the different viruses when the same virus input (5 ng/mL CA-p24) was used to infect TZM-bl cells. p24 concentration was measured by ELISA. Averages and standard errors from 2 independent experiments (each performed in duplicate) are shown and analyzed using unpaired t-test. **p < 0.005; ***p < 0.0005.

Removal of high-mannose glycans from HIV Env or enrichment of mannose-type glycans by using glycosylation biosynthetic inhibitors or cell lines was shown to reduce viral infectivity, while others has demonstrated increased infectivity with these same approaches [4, 6, 23]. Therefore, we investigated whether viruses produced under different glycosylation conditions would exhibit differential infectivity in TZM.bl reporter cells. Virus inputs were normalized based on p24 contents. The data shown in Fig 4A and 4B revealed that infectivity of JRFL and REJO viruses enriched with mannose-type glycans was diminished compared with infectivity of wild-type viruses: JRFL^KIF^ and REJO^KIF^ were least infectious, displaying less than 20% of their wild type counterparts, while infectivity of JRFL^GnTI-/-^, REJO^GnTI-/-^, and REJO^SWAIN^ was lower by ~50%. JRFL^SWAIN^ showed slight reduction in infectivity, but was significantly different from wild type (Fig 4B). These data illustrate that modulation of HIV-1 Env glycan composition affects virus infectivity.

### 3.4 *N*-glycan modification affects Env incorporation into viral particles

**Fig 5:**
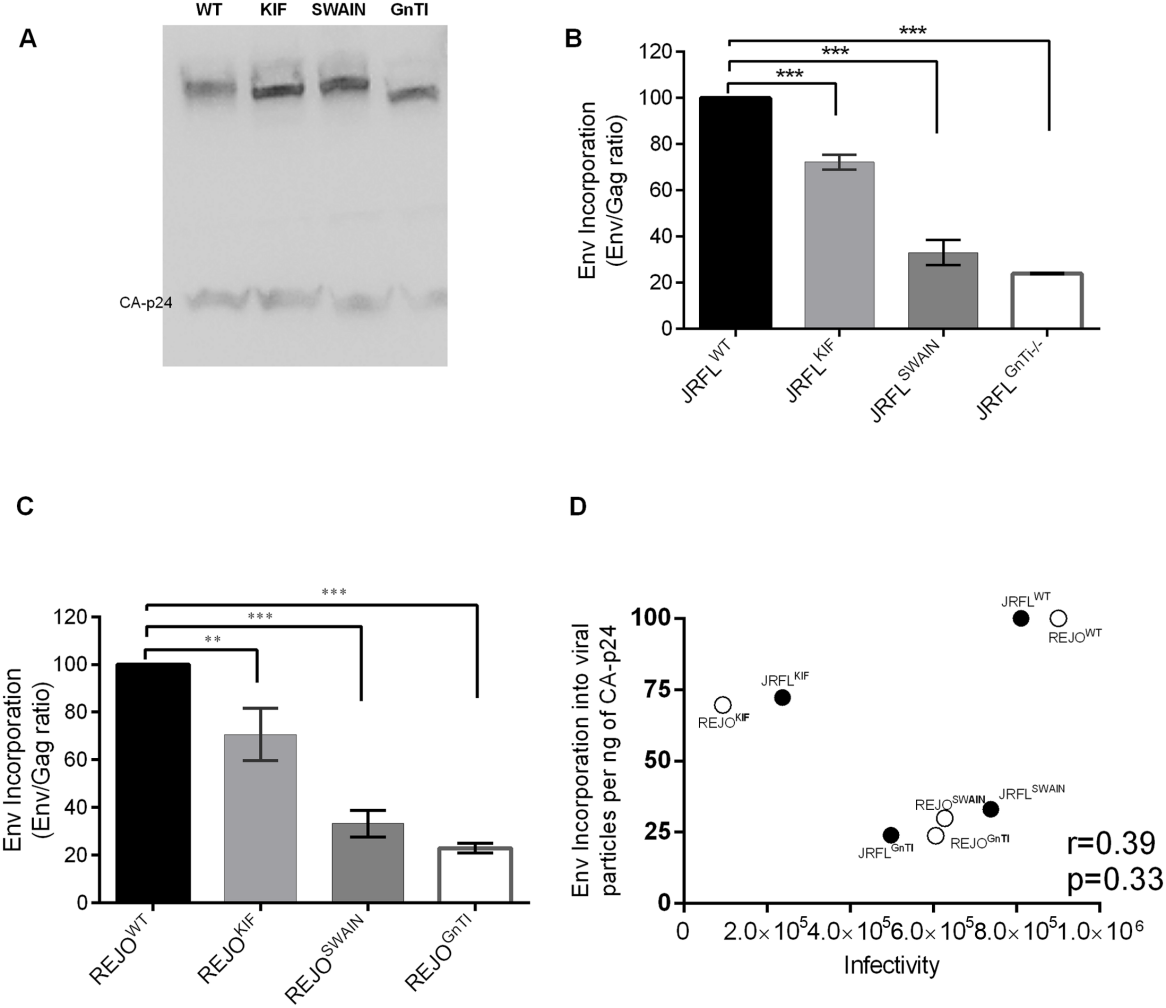
Effect of *N*-glycan modification on Env incorporation to virions. Viruses were produced in 293T HEK cells in the presence or absence of glycosidase inhibitors kifunensine and swainsonine or in 293S HEK GnTI-/- cells. A) JRFL virus preparations were loaded to SDS-PAGE after normalization for CA-p24 content. Env was detected by Western blot with anti-gp120 mAbs and quantified using Image Lab (BioRad) software. B) The relative contents of Env associated with N-glycan-modified JRFL viruses were calculated in comparison with Env of wild type control (set at 100%). C) The relative Env incorporation to N-glycan modified vs wild type REJO viruses. Data represent averages and standard errors from 2 experiments (each in duplicate) and were analyzed using unpaired t-test. ***p <0.0005, ** p <0.005. D) Correlation of Env incorporation to virions and virus infectivity by spearman’s rank test.

The reduced infectivity of viruses enriched with mannose-type glycans might be a result of lower expression of Env glycoprotein, a determining factor of virus infectivity. To investigate this, we measured Env incorporation in the virion by Western blot. The data in Fig 5A and 5B show changes in the amounts of Env incorporated into virus particles after glycan manipulation. Compared with JRFL^WT^, viruses with altered glycosylation had lower levels of Env incorporated into the virions in this order: JRFL^WT^ > JRFL^KIF^ > JRFL^SWAIN^ > JRFL^GnTI-/-^. Similar results were obtained with REJO (Fig 5C). Thus, enriching the glycan composition of viruses with mannose-type glycans reduced Env incorporation into virion particles, diminishing their infectivity. Indeed, a strong positive correlation was observed between viral infectivity and Env incorporation (p = 0.39; r = 0.33) (Fig 5D).

### 3.5 Increasing glycan homogeneity reduces differential sensitivity of viruses to lectins

**Fig 6:**
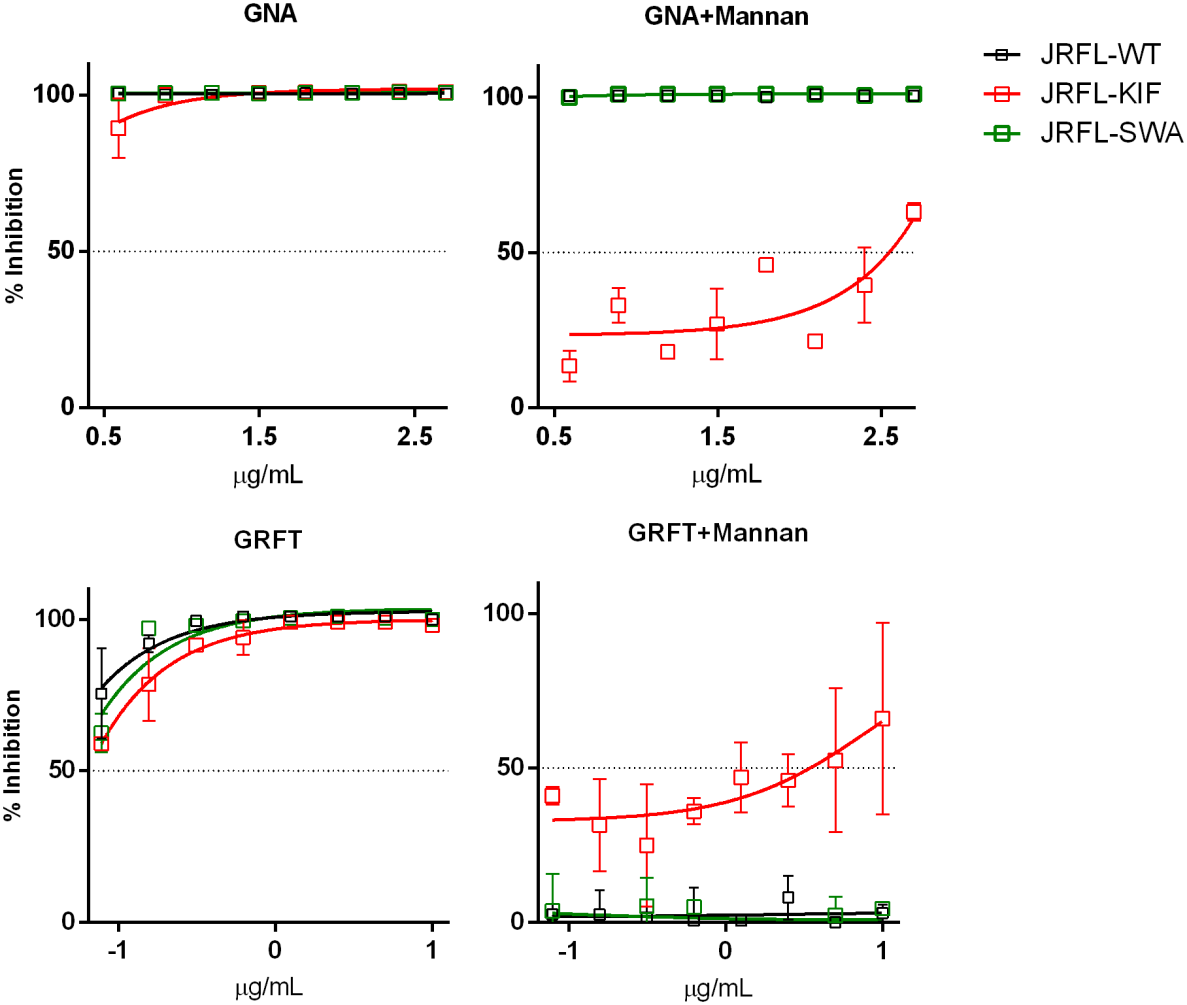
Mannan competition assay. GRFT and GNA were serially 2-fold diluted starting from 10μg and 250μg, respectively, to attain approximately 100% virus inhibition at all concentrations tested. When mannan (10 μg /mL) was used, it was added to lectins, and the mixtures were incubated with wild type or glycan-modified JRFL viruses (200 TCID_50_) for 1 h at 37°C, before addition of TZM-bl cells. After 48 h, virus infection was measured based on β-galactosidase activity. All experiments were performed in duplicate and repeated twice. Data are shown as averages and standard deviations from the 2 experiments.

To test the hypothesis that glycan heterogeneity contributes to the differential sensitivity of HIV-1 strains to lectins, we first examined whether the antivirus activity of lectins was mediated by lectin interaction with glycans on virus. To this end, we used mannan to compete for lectin binding and abrogate lectin-mediated virus inhibition. Mannan from *Saccharomyces cerevisiae*, which has mainly terminal α1-2Man residues, was used to block the inhibitory activity of GNA and GRFT, which recognize Manα1-3Man- and Manα1-2Man-terminal oligosaccharides present on Man_5-6_ and Man_7-9_ glycans, respectively. Serially diluted lectins were tested starting from a concentration of 10x IC_50_ in order to inhibit virus infection close to 100% in the absence of mannan (Fig 6). The *S*. *cerevisiae* mannan did not block GNA inhibition of JRFL^WT^ and JRFL^SWAIN^, reflecting the incompatible α1-2Man component of this mannan for GNA that preferential binds to Manα1-3Man-containing glycans. These results also indicate that JRFL^WT^ and JRFL^SWAIN^ viruses express high-mannose glycans of Man_5-9_GlcNAc_2_ and hybrid glycans which carry terminal Manα1-3Man residues. However, mannan partially affected GNA inhibition of JRFL^KIF^, and a similar effect was seen on GRFT inhibition of JRFL^KIF^, for reasons that remained unclear. In contrast, mannan completely abrogated the inhibitory effects of GRFT on JRFL^WT^ and JRFL^SWAIN^, consistent with the specificity of GRFT for Manα1-2Man-terminating glycans and the predominance of this particular terminal mannose moiety on the yeast mannan. These data provide evidence that that the antiviral activity of these lectins involves direct binding of the lectins to virus glycans.

**Fig 7:**
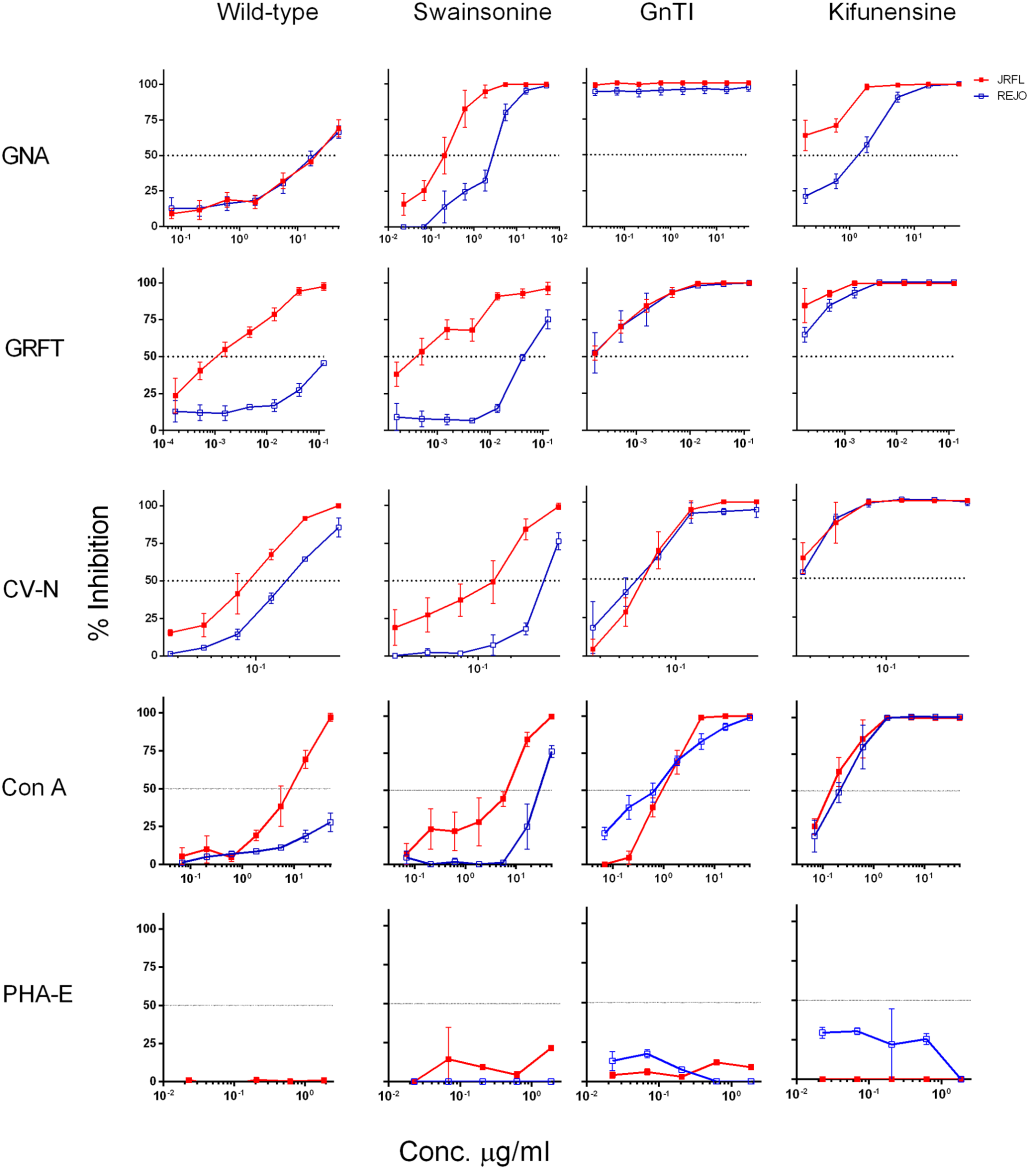
Effect of *N*-glycan modification on virus sensitivity to lectins. JRFL.JB and REJO4541.67 viruses were produced in 293T cells in the presence of glycosylation-pathway inhibitors kifunensine or swainsonine, or in 293S GnTI^-/-^ cells lacking GlcNAc transferase I. Lectins were serially diluted; viruses were then added at 200 TCID_50_ and incubated for 1 h at 37°C before addition of TZM-bl cells. After 48 h, virus infection was measured based on β-galactosidase activity. Cells infected with viruses only, in the absence of lectins, represent 100% infection. Percentage of virus inhibition by lectin was calculated using virus control (virus and cells only, 0% inhibition) and cell control (cell and no virus, 100% inhibition). All experiments were performed in duplicate and repeated three times. Data are shown as the averages and standard deviations from all 3 experiments.

Next, to assess whether differential sensitivity of HIV-1 viruses to inhibition by lectins was due to glycan heterogeneity on these viruses, JRFL and REJO viruses were produced in the presence of glycosidase inhibitors or in a mutant cell line as described above to express more homogenous glycans. Overall glycan-modified viruses, especially those produced in GnTI-/- cells and in kifunensine, were more sensitive to lectins than were wild type viruses (Table 3, Fig 7). Remarkably, the differential sensitivity of JRFL^WT^ and REJO^WT^ to GRFT (IC_50_ 0.001 vs 0.04 μg/mL) was completely eliminated, when the viruses were made in GnTI^-/-^ cells (IC_50_ <0.0001 μg/mL for both) or in kifunensine (IC_50_ 0.00001 vs 0.0001 μg/mL), but not in swansonine (IC_50_ 0.0003 vs 0.02 μg/mL). A similar pattern was seen with another α1,2 Man-specific lectin CV-N and the broadly reactive lectin Con-A. On the other hand, GNA (α1,3 Man-specific) and HHA (α1,6 Man-specific) showed a different pattern from GRFL, CV-N, and Con-A, while with PHA-E (complex glycan-specific), no inhibition was seen with wild type or glycan-modified viruses. Hence, by reducing glycan heterogeneity, JRFL and REJO viruses, which otherwise exhibited great differences in sensitivity to high mannose-specific lectins, became equally sensitive to these lectins. These data support the idea that the heterogeneity of glycans expressed by HIV-1 isolates contributes to their differential sensitivity to inhibitory activity of lectins.

**Table 3:** Effect of glycan modification of HIV-1 envelope on sensitivity to lectins.

		JRFL				REJO			
		WT	SWAIN	GnTI^-/-^	KIF	WT	SWAIN	GnTI^-/-^	KIF
	**GNA**	11.78	0.02	<0.00001	0.007	12.9	0.20	<0.00001	0.06
	**HHA**	>20	0.003	<0.00001	0.001	>20	6.55	<0.00001	2.09
	**GRFT**	0.001	0.0003	0.00018	<0.00001	0.04	0.02	0.0001	0.0001
	**CV-N**	0.09	0.11	0.07	<0.0001	0.24	0.75	0.03	<0.0001
	**Con A**	0.20	0.03	0.04	0.020	2.40	0.02	0.02	0.01
	**PHA-E**	>50	>50	>50	>50	>50	>50	>50	>50
	**<0.0001**		**0.001–0.01**		**0.01–0.1**		**0.1–10**		**>10**

IC_50_ values (μg/mL) of different lectins against glycan-modified HIV-1 pseudoviruses produced in kifunensine (KIF), swainsonine (SWAIN), or a cell line lacking GlcNAc I transferase I (GnTI^-/-^). Virus inhibition assay was performed in duplicate and repeated 3 times. Data are color-coded and shown as averages of all experiments.

### 3.6 Controlled enzymatic deglycosylation of HIV-1 Env

**Fig 8:**
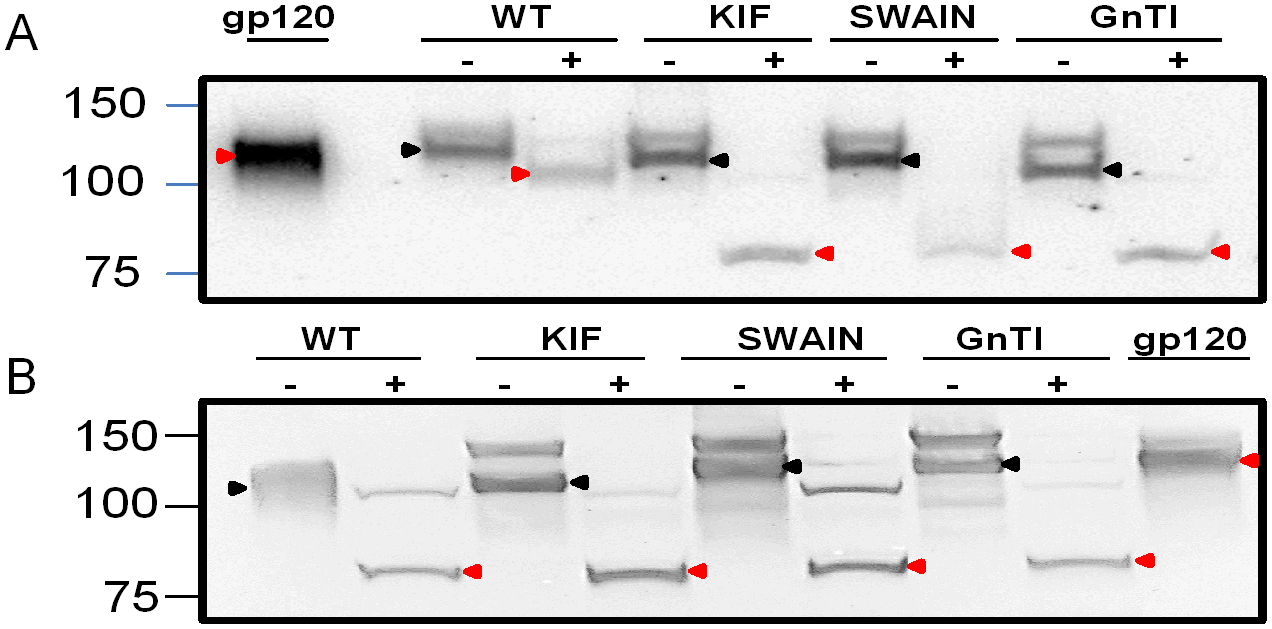
Deglycosylation of wild type vs glycan-modified Envs by Endo-H and PNGase F. a) JRFL viruses were produced in 293T cells with kifunensine or swansonine or in GnTI-/- cells, concentrated from culture supernatants by Lenti X-100, and then treated (+) or not treated (-) with Endo H to remove high-mannose and hybrid-mannose *N*-glycans but not complex *N*-glycans. b) JRFL viruses were similarly produced and treated with (+) or without (-) PNGase F to remove all *N*-glycans. The digestion products were subjected to SDS-PAGE under a reduced condition and Western blot analysis with anti-gp120 mAbs. Untreated Env gp120 and enzyme treated Env gp120 are indicated by black and red arrows, respectively.

To evaluate the relative abundance of different types of *N*-linked glycans present on Env of the different virus preparations, viruses were subjected to selective deglycosylation by endoglycosidase (Endo)-H, which cleaves in the *N*-glycan core between GlcNAcβ1-4GlcNAc of high-mannose and hybrid glycans, leaving complex glycans intact. Undigested Env from JRFL viruses produced with kifunensine or swainsonine, or in GnTI-/- cells displayed different migration patterns (Fig 8A), similar to the results in Fig 3. After digestion with Endo-H, wild type Env migrated slightly lower than undigested control, indicating significant retention of complex glycan after removal of high-mannose and hybrid glycans. In contrast, Env of viruses produced in kifunensine, swainsonine, or in GnTI-/- cells were completely sensitive to Endo H deglycosylation by Endo H, yielding protein bands of ~75KDa, which corresponds to the size of the gp120 protein backbone.

For comparison, these viruses were also digested with PNGase F, an amidase that cleaves between the innermost GlcNAc and asparagine residues and thereby removes all high-mannose, hybrid, and complex glycans from Env. The data shown in Fig 8B demonstrate that, for all viruses including wild type, PNGase digestion produced ~75kDa bands. Taken together, these results confirm that HIV-1 Env contained a heterogeneous mix of high-mannose, hybrid, and complex glycans, and this heterogeneity was diminished when the virus was produced in the presence of glycosidase inhibitors or in cells lacking a key enzyme in the glycosylation pathway.

## 4. Discussion

In this study, we focused primarily on how glycan heterogeneity or differences in patterns of *N*-linked glycans on HIV-1 Env glycoprotein determine the sensitivity of viruses to lectin-mediated inhibition. Taking into consideration the structure of Man_5-9_GlcNAc_2_ and the final maturation products of Man_5_GlcNAc_2_ into hybrid and complex glycans, we used mannose-binding lectins particularly that differentially recognize the different maturation products of the glycosylation biosynthetic pathway. Surprisingly, we found that different viruses—tier 1a and tier 1b viruses, which are highly sensitive to mAbs—were also highly sensitive to lectins, even though the lectins, unlike antibodies, do not interact with the protein component of their Env glycoprotein. All viruses tested were highly sensitive to Manα1-2Man-binding lectins GRFT, CV-N, and SV-N, compared with Manα1-3Man- and Manα1-6Man-binding lectins GNA and HHA; this is in accordance with the published literature [56–59]. It has been previously shown that the predominant glycans on HIV-1 Env are mostly oligomannose-containing glycans of Man_5-9_GlcNAc_2_ type, while a very small percentage of the total glycans on HIV-1 Env is represented by GlcNAcMan_5_GlcNAc_2_-derived (hybrid type) and GlcNAc_2_Man_5_GlcNAc_2_-derived (complex glycans) types of *N*-linked glycans [4, 26]. The reason we did not observe any inhibition with complex binding lectins like PHA-E or LCA is probably that the contribution of complex glycans to the viral Env may not be sufficient to inhibit viral infection.

We found a highly significant difference in sensitivity of JRFL and REJO viruses to lectins, with REJO displaying relatively less sensitivity than JRFL. In order to confirm our hypothesis that this differential sensitivity may be a result of glycan heterogeneity on Env of these viruses, we designed the strategy of inhibiting the glycosylation pathway using a combination of Mannosidase I, Mannosidase II, and GnTI^-/-^ defective cell line to limit the processing and maturation of Man_9_GlcNAc_2_ or Man_5_GlcNAc_2_, early precursors during glycan processing along the endoplasmic reticulum (ER) and Golgi [4, 60]. The viruses produced under these glycosylation inhibitory conditions displayed decreased molecular mass of Env in SDS-PAGE, consistent with the previous literature [30]. We also found that all viruses with altered glycan content showed progressively lower TCID_50_ values in TZM-bl cells compared with wild type, and viruses produced in GnTI^-/-^ cells showed lowest the value, while viruses produced in the presence of kifunensine and swainsonine were in the intermediate range. These viruses had a reduced infectious titer upon multiple freeze-thaw cycles, similar to that seen in wild-type viruses. Their infectivity in TZM-bl cells followed the same order and was in agreement with previous studies [6, 61]. However, other studies reported that the infectivity of HIV-1 in TZM-bl cells is not affected when viruses are produced in 293T cells in the presence of glycan-processing inhibitors kifunensine or GnTi^-/-^ cells [4, 30]. We also showed that Env incorporation into viral particles decreased under such conditions as compared with the wild type. These findings suggest that viruses with altered glycans produced under glycosylation-inhibitory conditions display reduced viral entry functions, infectivity, and Env incorporation, but viral fitness is not reduced to an extent that might affect their usefulness in studies of the antiviral properties of lectins.

Two of the viruses, JRFL (chronic) and REJO (acute), displayed highly significant differences in their sensitivity to lectins, with JRFL more sensitive to lectin inhibition than was REJO virus. In order to prove our hypothesis that this differential sensitivity may be a result of glycan heterogeneity on Env of these viruses, we designed the strategy of inhibiting the glycosylation pathway using combination of mannosidase I, mannosidase II, and GnTI-/- defective cell line to limit the processing and maturation of Man_9_GlcNAc_2_ or Man_5_GlcNAc_2_, early precursors during glycan processing along the ER and Golgi [4, 60]. The impact of Env glycan modification on the biological properties of virus has been examined previously, with inconsistent results. We found that viruses produced under glycosylation-inhibitory conditions displayed decreased molecular mass of Env in SDS-PAGE gels, consistent with the previous literature [30].

Glycan-modified viruses displayed a more homogenous and predictable pattern of glycosylation, with different degrees and extents of microheterogeneity and site occupancy. Viruses tested with GNA and HHA, which recognize Manα1-3Man and Manα1-6Man, respectively, inhibited both JRFL and REJO viruses differentially. However, enrichment of oligomannose-containing glycans on viruses resulted in increased sensitivity of viruses to GNA and HHA. A similar trend was observed with other mannose-binding lectins. The higher sensitivity of glycan-modified viruses to mannose-binding lectins can be explained by the fact that enrichment of Man_5-9_GlcNAc_2-_ and GlcNAcMan_5_GlcNAc_2_-derived hybrid glycans results in a preponderance of Manα1-3Man-, Manα1-2Man-, and Manα1-6Man-terminating glycans on the viral Env, which provides more optimal interaction between the virus Env and mannose-binding lectins. The high sensitivity of GnTI-/- viruses to GNA and HHA can be attributed to an abundance of Man_5_GlcNAc_2_ glycans with termini exclusively having Manα1-3Man and Manα1-6Man glycan structures. Further, the viruses produced in the presence of swainsonine were more sensitive to Manα1-3Man- and α1-6Man-binding lectins than those produced in the presence of kifunensine; this can be explained by the enrichment of Man_5-9_GlcNAc_2_ and hybrid (GlcNAcMan_5_GlcNAc_2_-derived) glycans, which present a greater abundance of Manα1-3Man- and Manα1-6Man-terminating glycans. Viruses produced in the presence of kifunensine express predominantly Man_9_GlcNAc_2_ glycans, whose branches terminate with Manα1-2Man structures. However, the higher sensitivity of kifunensine viruses compared with wild-type viruses can be explained by the fact that kifunensine does not exclusively produce Man_5_GlcNAc_2_ glycoforms but also produces Man_5-9_GlcNAc_2_ [4, 62]. Similarly, both JRFL and REJO, when produced under glycosylation-inhibitory conditions, were equally sensitive to Manα1-2Man-binding lectins GRFT and SV-N, the former being more potent than the latter, in agreement with previously published literature [45, 46, 63]. Although there were clear differences in inhibition between wild type JRFL and REJO, both viruses were equally sensitive when produced in the presence of kifunensine, swainsonine, and GnTI^-/-^, due to the progressively reduced content of Manα1-2Man, which is exclusively present on the terminal branches of Man_9_GlcNAc_2_. The content of Man_9_GlcNAc_2_ is highest with kifunensine treatment followed by Man_5-9_GlcNAc_2_, GlcNAcMan_9_GlcNAc_2_, and Man_5_GlcNAc_2_, produced in the presence of swainsonine and GnTI^-/-^, respectively. These results of differential inhibition to lectins correlate strongly with glycan heterogeneity and sensitivity with progressively increasing glycan homogeneity.

We also tested Con-A a nonspecific lectin, with a binding affinity highest for Man α [1–3]-[Man α-[1–6]] Man trisaccharide core of *N*-glycan, but it can recognize all forms of NLGs. Both REJO and JRFL glycan-defective viruses were equally sensitive, with no significant difference, indicating that the relative resistance displayed by REJO compared with JRFL virus might be a result of more complex glycans being present on REJO or more oligomannose being present on the JRFL virus. However, no inhibition was observed by complex glycan binding lectins PHA-E and LCA, which recognize Galβ1-4GlcNAcβ1-2Man and α (1–6) linked fucosylated NLGs, respectively, indicating that the complex glycans are relatively scarce on the surface of HIV-1, in agreement with previously published results [4, 26, 56].

The wild-type viruses were relatively resistant to Endo H glycosidase digestion, while viruses produced under different glycosylation inhibitory conditions deglycsoylated completely. These findings indicated that the wild-type virus has a heterogeneous glycan composition, including complex glycans, thus rendering it resistant to Endo H deglycosylation. HIV-1 viruses with progressively more oligomannose and less or absent complex glycans display an enhanced sensitivity to Endo H deglycosylation. In contrast to Endo H deglycosylation, when the viruses were deglycosylated with PNGase F, all the viruses behaved similarly, thus providing evidence that the resistant nature of wild-type viruses with Endo H resulted in glycan heterogeneity of the viruses.

In summary, HIV-1 Env glycan patterns are diverse among different viruses. These heterogeneous glycans are responsible for the differential sensitivity of the viruses to lectins that are being investigated for the development of anti-HIV microbicides. In addition to helping in understanding the relative glycan heterogeneity of different HIV-1 strains and dependence of lectins on glycan composition, our results may have far-reaching implications for the better design and selection of anti-HIV-1 lectins.
